# *Botrytis cinerea* G Protein β Subunit Bcgb1 Controls Growth, Development and Virulence by Regulating cAMP Signaling and MAPK Signaling

**DOI:** 10.3390/jof7060431

**Published:** 2021-05-29

**Authors:** Jiejing Tang, Mingde Wu, Jing Zhang, Guoqing Li, Long Yang

**Affiliations:** State Key Laboratory of Agricultural Microbiology and Hubei Key Laboratory of Plant Pathology, Huazhong Agricultural University, Wuhan 430070, China; tangjiejing@webmail.hzau.edu.cn (J.T.); mingde@mail.hzau.edu.cn (M.W.); zhangjing1007@mail.hzau.edu.cn (J.Z.); guoqingli@mail.hzau.edu.cn (G.L.)

**Keywords:** *Botrytis cinerea*, Gβ subunit, cAMP signaling pathway, MAPK signaling pathway

## Abstract

*Botrytis cinerea* is a necrotrophic phytopathogenic fungus that causes gray mold disease in many crops. To better understand the role of G protein signaling in the development and virulence of this fungus, the G protein β subunit gene *Bcgb1* was knocked out in this study. The Δ*Bcgb1* mutants showed reduced mycelial growth rate, but increased aerial hyphae and mycelial biomass, lack of conidiation, failed to form sclerotia, increased resistance to cell wall and oxidative stresses, delayed formation of infection cushions, and decreased virulence. Deletion of *Bcgb1* resulted in a significant reduction in the expression of several genes involved in cAMP signaling, and caused a notable increase in intracellular cAMP levels, suggesting that G protein β subunit Bcgb1 plays an important role in cAMP signaling. Furthermore, phosphorylation levels of MAP kinases (Bmp1 and Bmp3) were increased in the Δ*Bcgb1* mutants. Yeast two-hybrid assays showed that Bcgb1 interacts with MAPK (Bmp1 and Bmp3) cascade proteins (BcSte11, BcBck1, BcMkk1, and BcSte50), and the Bmp1-regulated gene *Bcgas2* was up-regulated in the Δ*Bcgb1* mutant. These results indicated that Gβ protein Bcgb1 is involved in the MAPK signaling pathway in *B. cinerea*. In summary, our results revealed that Gβ protein Bcgb1 controls development and virulence through both the cAMP and MAPK (Bmp1 and Bmp3) signaling pathways in *B. cinerea*.

## 1. Introduction

*Botrytis cinerea* is an important phytopathogenic fungus and the causal agent of gray mold disease in more than 1400 plant species. It is responsible for significant economic losses in many important vegetables, fruits, and ornamentals [1]. The cost of controlling gray mold disease in the world has been estimated at over €1 billion per year. Due to its scientific and economic importance, *B. cinerea* is considered as the second most important fungal pathogen and the necrotrophic model fungus [2]. In the life cycle of *B. cinerea*, there are four different structures, including conidia, mycelia, sclerotia, and ascospores. Since sexual ascospores rarely occur in nature, the main source of the initial inoculum in the field is asexual conidia that formed from germinating sclerotia or hyphae, or survived in the last season [3]. Sclerotia are the melanized dormancy structures that can survive in adverse environment. When favorable conditions appear in spring, sclerotia will germinate to produce hyphae and conidia as the source of initial infection. Therefore, sclerotia and conidia play pivotal roles in the epidemic and life cycle of *B. cinerea* [3].

Heterotrimeric G proteins, which consist of Gα, Gβ, and Gγ subunits, transmit a variety of extracellular signals received by membrane-spanning G protein coupled receptors (GPCR) to intracellular effectors of eukaryotic cells [4]. When GPCR senses external signal stimulation, it triggers GDP-GTP exchange in Gα, leading to the dissociation of G protein complex as Gα-GTP and Gβγ dimer. Both Gα-GTP and Gβγ dimer can activate and regulate downstream signaling pathways, such as the cAMP and MAP kinase pathways [4,5].

In filamentous fungi, G proteins have been demonstrated to be required for growth, differentiation, mating, sporulation, and pathogenesis [4]. Like most characterized filamentous fungi, the plant pathogen *Magnaporthe oryzae* contains three Gα subunit genes (*magA*, *magB*, and *magC*); one Gβ subunit gene, *mgb1*; and one Gγ subunit gene, *MGG1*. Three Gα subunit genes are involved in *M. oryzae* mating, but only *magB* (group I), like *mgb1* and *MGG1*, is required for appressorium formation and virulence [6,7,8]. In the soilborne vascular wilt fungus *Fusarium oxysporum*, deletion mutants of the Gα subunit Fga1 (group I) and the Gβ subunit Fgb1 displayed the similar phenotypes, i.e., altered colony morphology, reduced virulence and conidiation, and increased heat resistance [9,10]. However, deletion of the group III Gα subunit Fga2 results in some phenotypes different than those of Fga1 and Fgb1 mutants, such as complete loss of pathogenicity, no alteration on colony morphology, and conidiation [11]. Mutants lacking Fga1 or Fgb1 exhibit reduced intercellular cAMP levels, suggesting that Gα subunit Fga1 and Gβ subunit Fgb1 are involved in the cAMP signaling pathway [9,10]. In another soilborne vascular wilt fungus, *Verticillum dahliae*, the Gβ subunit gene *VGB* positively regulates virulence and negatively regulates conidiation and microsclerotia formation [12].

In *B. cinerea*, the function of three Gα subunit genes (*Bcg1*, *Bcg2*, and *Bcg3*) has been demonstrated by targeted gene deletion, suggesting that all of them are involved in the infection process. The *Bcg1* deletion mutants show altered colony morphology and significantly reduced virulence [13,14]. In contrast, deletion of *Bcg2* only results in a slight decrease in pathogenicity [13]. The third Gα subunit, Bcg3, is important for conidiation, conidial germination, and virulence [15]. Δ*Bcg1* and Δ*Bcg3* mutants show reduced intercellular cAMP levels and their defects are partially restored by exogenous cAMP, implying that Bcg1 and Bcg3 are the upstream components of cAMP signaling pathways. Although the Gα subunits of *B. cinerea* have been investigated comprehensively, the functional role of the Gβ subunit in growth, conidiation, sclerotia formation, and pathogenicity, as well as its downstream signaling pathway in *B. cinerea*, is still unclear.

In this study, we knocked out the Gβ subunit gene *Bcgb1* using the split-marker strategy. The Δ*Bcgb1* mutants exhibited defects in mycelial growth, conidiation, sclerotia formation, and virulence. Deletion of *Bcgb1* affected intracellular cAMP levels and the phosphorylation level of MAP kinases (Bmp1 and Bmp3). Yeast two-hybrid assays showed that Bcgb1 directly interacts with Bmp1 upstream kinase BcSte11, Bmp3 upstream kinases BcBck1 and BcMkk1, and the BcSte11/BcSte7/Bmp1 MAP kinase adaptor protein BcSte50. Moreover, the qRT-PCR result showed that *Bcgas2*, the downstream target gene of Bmp1 [16], was remarkably up-regulated in Δ*Bcgb1*. These results suggest that Gβ protein Bcgb1 is involved in regulation of the development and virulence via both cAMP signaling and MAPK (Bmp1 and Bmp3) signaling in *B. cinerea*.

## 2. Materials and Methods

### 2.1. Fungal Strains and Culture Conditions

The wild-type strain B05.10 and its derived strains, including *Bcgb1* gene knockout mutants (Δ*Bcgb1*-8, Δ*Bcgb1*-43, and Δ*Bcgb1*-64), were cultivated on potato dextrose agar (PDA) [17] at 20 °C. The *Bcgb1* gene knockout mutants were maintained on PDA amended with 100 μg·mL^−1^ hygromycin B (Calbiochem, San Diego, CA, USA). For growth experiments, the mutants and B05.10 were grown on PDA at 20 °C. Each plate was inoculated with a 5 mm-diameter mycelial agar plug taken from the edge of a 2-day-old colony. To characterize the growth rate, sclerotia formation, and infection cushion formation, a different strain was cultured in constant darkness. To characterize the sporulation, strains were grown under a 12 h light/dark cycle. To test the mycelial biomass, 10 mycelial plugs (5 mm) of each strain were inoculated into an Erlenmeyer flask (250 mL) containing 100 mL potato dextrose broth (PDB) [17], with three flasks for each strain, and the flasks were shake-incubated at 20 °C and 150 rpm for 2 days. Mycelial biomass of each strain was harvested by paper-filtering, dried at 55 °C for 12 h, and weighed. To evaluate the response of *Bcgb1* knockout mutants to abiotic stress, the wild-type strain and *Bcgb1* knockout mutants were cultured on PDA medium amended with 1 M NaCl, 1 M KCl, 1 M sucrose, 1 M sorbitol, 0.1 mg/mL SDS, 0.3 mg/mL Congo Red (CR), 0.2 mg/mL CalcoFluor White (CFW), and 5mM H_2_O_2_. The colony diameters were measured at 72 h to calculate the relative mycelial growth rate of each strain. Each experiment was repeated three times.

### 2.2. Disruption of Bcgb1

The *Bcgb1* gene was disrupted using the split marker method [18]. The disruption strategy for *Bcgb1* is showed in Figure S1. The 5′ and 3′ flanking sequences of *Bcgb1* were amplified with the primers listed in Table S1 and then fused with part of the hygromycin fragment. Two split-marker DNA fragments were transformed into protoplasts of the WT strain B05.10 using the PEG-mediated transformed technique [19]. The hyphal tips of the deletion transformants were screened on PDA plates containing hygromycin B (100 μg mL^−1^) three times and verified by PCR. Single spore isolation was performed to obtain the homokaryotic deletion mutants. Three *Bcgb1* deletion mutants, Δ*Bcgb1*-8, Δ*Bcgb1*-43, and Δ*Bcgb1*-64, were further confirmed by Southern blot analysis using the right flank of the *Bcgb1* gene as a probe. Southern blot analysis was performed by the Gene Images^TM^ AlkPhos Direct^TM^ labeling and detection kit from GE Healthcare (Amersham Biosciences, Buckinghamshire, UK).

### 2.3. Extraction of DNA and RNA

Strains of *B. cinerea* were grown on PDA medium at 20 °C under darkness for 2 days. Genomic DNA of *B. cinerea* was extracted from the mycelia using the CTAB method [20]. Total RNA was extracted from mycelium samples of *B. cinerea* using the RNAiso Plus reagent (TaKaRa, Dalian, China) according to the manufacturer’s instructions.

### 2.4. Pathogenicity and Penetration Assays

A pathogenicity test was performed with 5-week-old tobacco (*Nicotiana benthamiana*) leaves using 5 mm mycelial plugs from wild-type, Δ*Bcgb1*-8, and Δ*Bcgb1*-43 mutant strains grown on PDA. Infected leaves were incubated at 20 °C under darkness with 100% relative humidity. The lesion diameters were measured at 72 h post inoculation.

Infection cushions were observed on onion epidermis as per a previous study [21]. Mycelial plugs (5 mm) of each strain were inoculated on onion epidermis and incubated at 20 °C under darkness. The epidermis was sampled and then stained with cotton blue before microscopic examination at 12 h and 24 h post inoculation, respectively. Each experiment was repeated three times.

### 2.5. Quantification of Intracellular cAMP

Mycelia were harvested from two-day-old PDB [17] liquid cultures, frozen in liquid nitrogen, and lyophilized for 20 h. For every 10 mg of lyophilized mycelium, 1 mL of 0.1 M HCl was added. Samples were centrifuged at 12,000 rpm for 15 min. The supernatant was used to determine cAMP concentration via the Monoclonal Anti-cAMP Antibody Based Direct cAMP ELISA Kit (NewEast Biosciences, Malvern, PA, USA) following the manufacturer’s instructions.

### 2.6. Reverse Transcription and Fluorescence Quantitative PCR (RT-qPCR)

The cDNA was synthesized via the PrimeScript^TM^ RT reagent kit (TaKaRa, Dalian, China) according to instructions from the manufacturer. An RT-qPCR was carried out in a CFX96 real-time PCR system (Bio-Rad, Hercules, CA, USA) with TB Green ^®^ Premix Ex Taq™ (Tli RNaseH Plus) (TaKaRa, Dalian, China). The *B. cinerea* actin gene *BcactA* (*Bcin16g02020*) was used as internal control. The relative expression of each gene was evaluated using the ^ΔΔ^*C*T method [22]. All primers used for the RT-qPCR analyses are listed in Table S1. The RT-qPCR assay was repeated three times, each with three biological replicates.

### 2.7. Assays for Bmp1 and Bmp3 Phosphorylation

Total proteins were isolated from two-day-old mycelia with the protein lysis buffer (50 mM Tris-HCl, pH 7.4, 150 mM NaCl, 1 mM EDTA, 1% Triton X-100) containing 1% each of protease inhibitor cocktail, phosphatase inhibitor cocktail 2, and phosphatase inhibitor cocktail 3 (Sigma-Adrich, St. Louis, MO, USA) as previously described [23]. Then, the total proteins were separated by 10% SDS-PAGE and then transferred to PVDF (polyvinylidene difluoride) membranes (Bio-Rad, Hercules, CA, USA). Phosphorylation of the Bmp1 and Bmp3 MAP kinases was detected by using the phospho-p44/42 MAPK antibody (Cell Signaling Technology, Boston, MA, USA). The total Bmp1 and Bmp3 was detected with anti-MAPK ERK 1/2 antibody (Santa Cruz Biotechnology, Dallas, TX, USA). The anti-GAPDH was used as a loading control.

### 2.8. Yeast Two–Hybrid Assays

The Matchmaker^TM^ Gold yeast two-hybrid system (Clontech, Mountain View, CA, USA) was used to analyze the protein–protein interactions. The full-length cDNA of *Bcgb1* (*Bcin08g01420*) was cloned into pGADT7 vector. Full-length cDNAs of *BcSte11* (*Bcin03g02630*), *BcSte7* (*Bcin04g05630*), *BcBck1* (*Bcin02g06590*), *BcMkk1* (*Bcin03g07190*), and *BcSte50* (*Bcin08g03660*) were cloned into pGBKT7 vector. A pair of plasmids (pGBKT7-53 and pGADT7-T) served as a positive control and a pair of plasmids (pGBKT7-Lam and pGADT7-T) was used as a negative control. The resulting prey and bait constructs were co-transformed in pairs into yeast strain Y2H following the manufacturer’s instructions. Transformants were grown on SD-Leu-Trp at 30 °C for 3 days, and then transferred to SD-His-Leu-Trp. The resulting yeast cells were further tested for β-galactosidase activities. The primers used in this experiment are listed in Table S1.

## 3. Results

### 3.1. Identification and Deletion of the Bcgb1 Gene in B. cinerea

**Figure 1:**
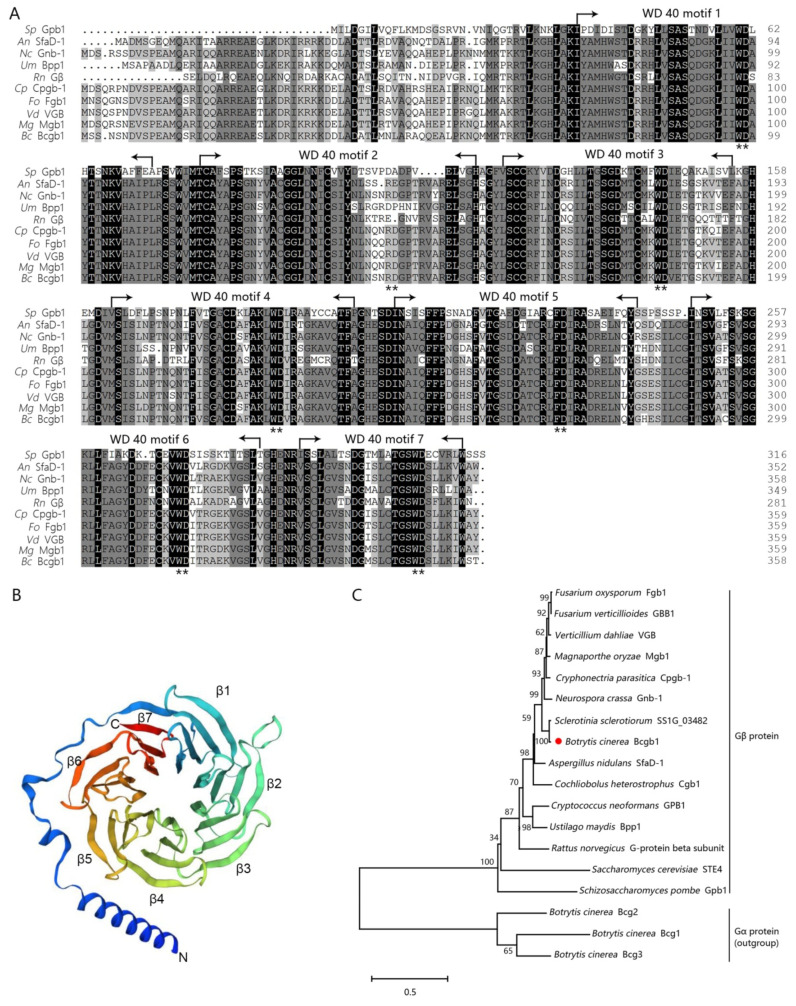
Sequence analysis of Bcgb1 in *B. cinerea*. (**A**) Amino sequence alignment of Bcgb1 orthologues. All conversed residues are shown in black and similar residues in grey. The positions of the seven WD repeats are labeled and indicated by arrows. ** means the conserved WD residues in WD repeats. (**B**) The crystal model of Bcgb1. The Gβ protein of *R. norvegicus* Gβ (PDB: 7cfm.1.B) was used as the template for the Bcgb1 model at the SWISS-MODEL website. The seven β-propeller blades were numbered. (**C**) A neighbor-joining tree based on amino acid sequences of Gβ protein in fungi. The following protein sequences were used: XP_018249805 (fgb1), ABE67098 (GBB1), XP_028497849 (VGB), BAC01165 (mgb1), XP_009851210 (gnb-1), XP_024550185 (Bcgb1), XP_001595393 (SS1G_03482), XP_024345016 (SfaD), XP_657685 (sfaD-1), AAO25585 (cgb1), AAD03596 (GPB1), XP_011386498 (bpp1), 5TDH_B (*R. norvegicus* Gβ protein), NP_014855 (STE4), AAC37501 (gpb1), XP_024548939 (Bcg1), XP_024552854 (Bcg2), and XP_024553380 (Bcg3). Bootstrap values (%) from 1000 replicates of the data are indicated above the nodes. The red dot represents the Gβ protein Bcgb1 of *B. cinerea* in this study.

The *Bcgb1* gene (*Bcin08g01420*) encoded a conserved Gβ subunit protein. The Bcgb1 protein (358 amino acids) contained a 7-WD40 repeat domain and shared high amino acid sequence identity with Gβ proteins in *Aspergillus nidulans* (82.12%), *Neurospora crassa* (88.83%), *Ustilago maydis* (67.6%), *Rattus norvegicus* (64.53%), *Cryphonectria parasitica* (88.58%)*, F. oxysporum* (89.69%), *V. dahliae* VGB (88.86%), and *M. oryze* (88.86%), although had only 37.5% identity with the Gβ subunit gpb1 in *Schizosaccharomyces pombe* (Figure 1A). Moreover, phylogenetic analysis also showed that Bcgb1 belonged to the same cluster as the Gβ proteins previously reported using Gα proteins as the outgroup (Figure 1C). Among the known Gβ protein in the Protein Data Bank (PDB), the crystal structure of Bcgb1 was predicted by using *R. norvegicus* Gβ protein (PDB: 7cfm.1.B) as a template (Figure 1B).

To investigate the function of Bcgb1, a Δ*Bcgb1* knockout mutant was generated by replacing the *Bcgb1* gene with a hygromycin-resistance cassette (HPT) (Figure S1A). After PEG-mediated transformation, three Δ*Bcgb1* mutants (Δ*Bcgb1*-8, Δ*Bcgb1*-43, and Δ*Bcgb1*-64) were obtained through PCR verification (Figure S1B) and further confirmed by Southern blotting analysis (Figure S1C).

### 3.2. Bcgb1 Is Required for Hyphal Growth, Conidiation, Sclerotia Formation

**Figure 2:**
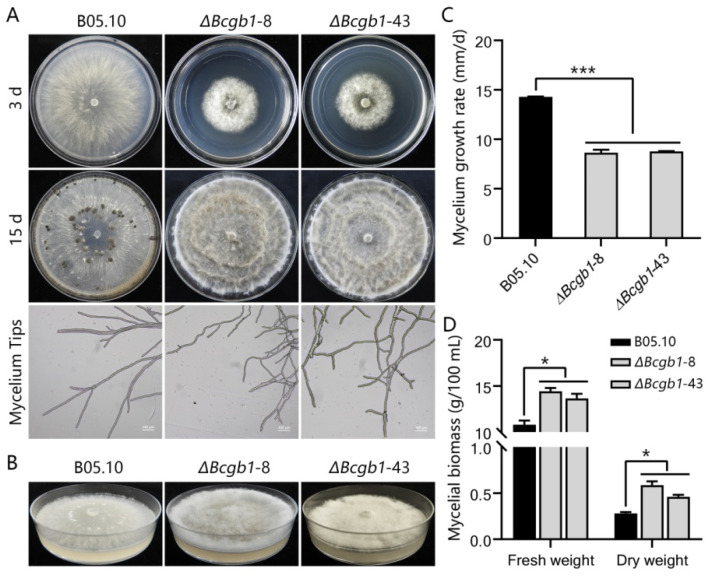
Bcgb1 is required for mycelial growth, conidiation, and sclerotia formation. (**A**) Colony morphology (3 d and 15 d) and mycelium tips (48 h) of the indicated strains cultured on PDA at 20 ℃. (**B**) Aerial hyphae growth is increased in the Δ*Bcgb1* mutants after incubation on PDA for 7 days at 20 °C. (**C**) Mycelial growth rate of the indicated strains cultured on PDA at 20 °C *** *p* < 0.001. (**D**) Mycelial biomass of the indicated strains cultured in PDB at 20 °C for 2 d. * *p* < 0.05.

To determine the role of Bcgb1 in hyphal growth, conidiation, and sclerotia formation, two Δ*Bcgb1* mutants (Δ*Bcgb1*-8 and Δ*Bcgb1*-43) grown on PDA were compared with the wild-type strain B05.10. Colonies of Δ*Bcgb1* mutants showed a fluffy, dense aspect; a decreased colony diameter; and dramatically increased aerial hyphae compared to the wild type (Figure 2A,B). Microscopic analysis showed that the Δ*Bcgb1* mutants produced more branches at the tip of the hyphae than that of the wild type (Figure 2A). After 15 days of incubation on PDA, the wild-type strain produced a large number of conidia and formed sclerotia. However, the Δ*Bcgb1* mutants were unable to produce conidia and sclerotia (Figure 2A). In comparison with the wild type, the mycelial growth rate of the Δ*Bcgb1* mutants was significantly reduced (Figure 2C), but the mycelial biomass was increased (Figure 2D). These results indicate that Bcgb1 plays an important role in hyphal growth, conidiation, and sclerotia formation.

### 3.3. Bcgb1 Is Involved in Response to Cell Wall and Oxidative Stresses

**Figure 3:**
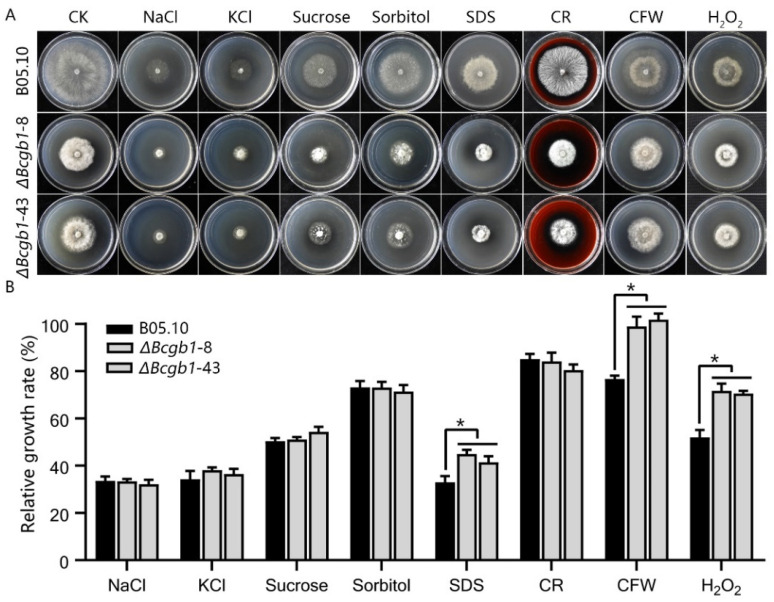
Bcgb1 is involved in responses to cell-wall and oxidative stresses. (**A**) Sensitivity test of strains to salt stress (NaCl or KCl), osmotic stress (sucrose or sorbitol), cell-wall stress (SDS, CR, or CFW), and oxidative stress (H_2_O_2_). Strains were incubated on PDA supplemented with 1 M NaCl, 1 M KCl, 1 M sucrose, 1 M sorbitol, 0.1 mg/mL SDS, 0.3 mg/mL CR, 0.2 mg/mL CFW, and 5 mM H_2_O_2_ at 20 °C for 72 h. (**B**) The relative mycelial growth rate of the indicated strains in the presence of various stresses. * *p* < 0.05.

To investigate functions of Bcgb1 in cell-wall integrity, we examined the sensitivity of the Δ*Bcgb1* mutants to osmotic stress agents NaCl, KCl, sucrose, and sorbitol; cell-wall disturbing agents SDS, CR, and CFW; and oxidative stress H_2_O_2_. Our results show that there was no significant difference in relative growth rate between the Δ*Bcgb1* mutants and wild type when cultured on PDA containing NaCl, KCl, sucrose, sorbitol, and CR (Figure 3). However, the relative growth rate of the Δ*Bcgb1* mutants significantly increased when cultured on PDA containing SDS, CFW, and H_2_O_2_ (Figure 3A,B). These results indicate that Bcgb1 plays a role in response to cell-wall and oxidative stresses.

### 3.4. Bcgb1 Is Important for Virulence in B. cinerea

**Figure 4:**
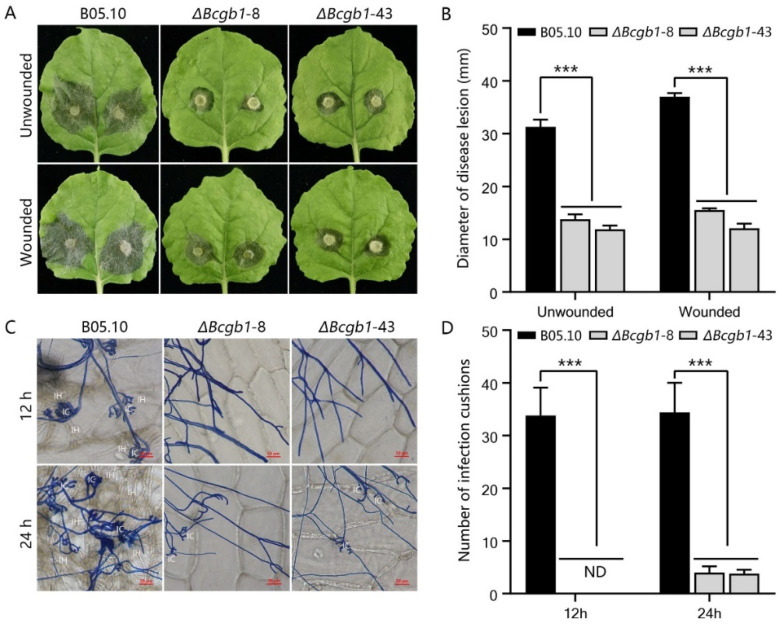
Bcgb1 is important for virulence in *B. cinerea*. (**A**) Pathogenicity test of the indicated strains on unwounded and wounded tobacco leaves. Disease symptoms were photographed at 72 h post inoculation (20 °C). (**B**) Lesion size caused by the indicated strains in A. (**C**) Infection cushion formation by mycelium plugs of the indicated strains on onion epidermis at 12 h or 24 h post inoculation (20 °C). IC: infection cushion, IH: infectious hyphae. (**D**) Quantitative analysis of infection cushions of the indicated strains in C. *** *p* < 0.001.

To analyze the role of Bcgb1 in pathogenicity, unwounded and wounded tobacco leaves were inoculated with the mycelial agar plugs of Δ*Bcgb1* mutants. The Δ*Bcgb1* mutants showed significantly reduced virulence in tobacco leaves (Figure 4A). At 72 hpi, the lesion size of Δ*Bcgb1* mutants on both unwounded and wounded leaves decreased by more than 50% compared with that of the wild type (Figure 4B). To determine the virulence defects of Δ*Bcgb1* mutants in detail, we performed a penetration assay on onion epidermis. As show in Figure 4C, the wild-type strain formed numerous infection cushions and successfully penetrated onion cells at 12 hpi and 24 hpi. However, the average number of infection cushions of Δ*Bcgb1* mutants was much less than that of the wild type (Figure 4D). This revealed that the Δ*Bcgb1* mutants delayed the formation of infection cushions to penetrate plant cells, resulting in the decrease of virulence. These results show that Bcgb1 is important for infection cushion formation and virulence.

### 3.5. Bcgb1 Is Involved in the Regulation of Intracellular cAMP Levels

**Figure 5:**
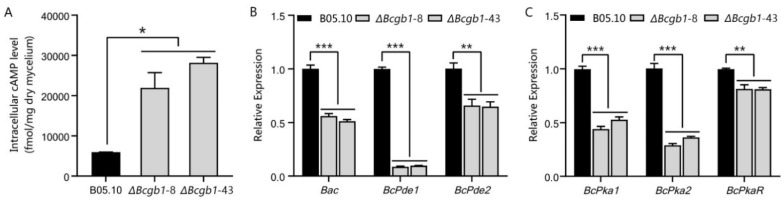
Bcgb1 is involved in the regulation of intracellular cAMP levels. (**A**) Quantitative determination of intracellular cAMP levels in mycelia of the indicated strains cultured in PDB for 2 days. Two biological repetitions with three replicates were assayed. The error bars represent the SD of three replicates. (**B**) Transcript level of *Bac*, *BcPde1*, and *BcPde2* in the WT and the Δ*Bcgb1* mutants of *B. cinerea*. (**C**) Transcript level of *BcPka1*, *BcPka2*, and *BcPkaR* in the WT and the Δ*Bcgb1* mutants of *B. cinerea*. * *p* < 0.05, ** *p* < 0.01, *** *p* < 0.001.

To test whether deletion of Bcgb1 affects the cAMP levels in *B. cinerea*, the intracellular cAMP levels were measured in the hyphae stage of the Δ*Bcgb1* mutants and wild type. The cAMP levels of two Δ*Bcgb1* mutants were drastically increased about fourfold and sixfold, respectively, compared to the wild type (Figure 5A).

Due to the cAMP levels having increased in Δ*Bcgb1* mutants, we further examined the transcript levels of the cAMP signaling pathway-related genes, such as the adenylate cyclase gene *Bac*, two phosphodiesterase genes (*BcPde1* and *BcPde2*), and three cAMP-dependent protein kinase (PKA) encoding genes (*BcPka1*, *BcPka2*, and *BcPkaR*). Interestingly, the expression of these six genes (*Bac*, *BcPde1*, *BcPde2*, *BcPka1*, *BcPka2*, and *BcPkaR*) was all significantly reduced in the Δ*Bcgb1* mutants (Figure 5B,C). These results indicate that Bcgb1 is required for maintaining normal cAMP levels in *B. cinerea*.

### 3.6. Bcgb1 Plays an Important Role in Two MAPK (Bmp1 and Bmp3) Signaling Pathways

**Figure 6:**
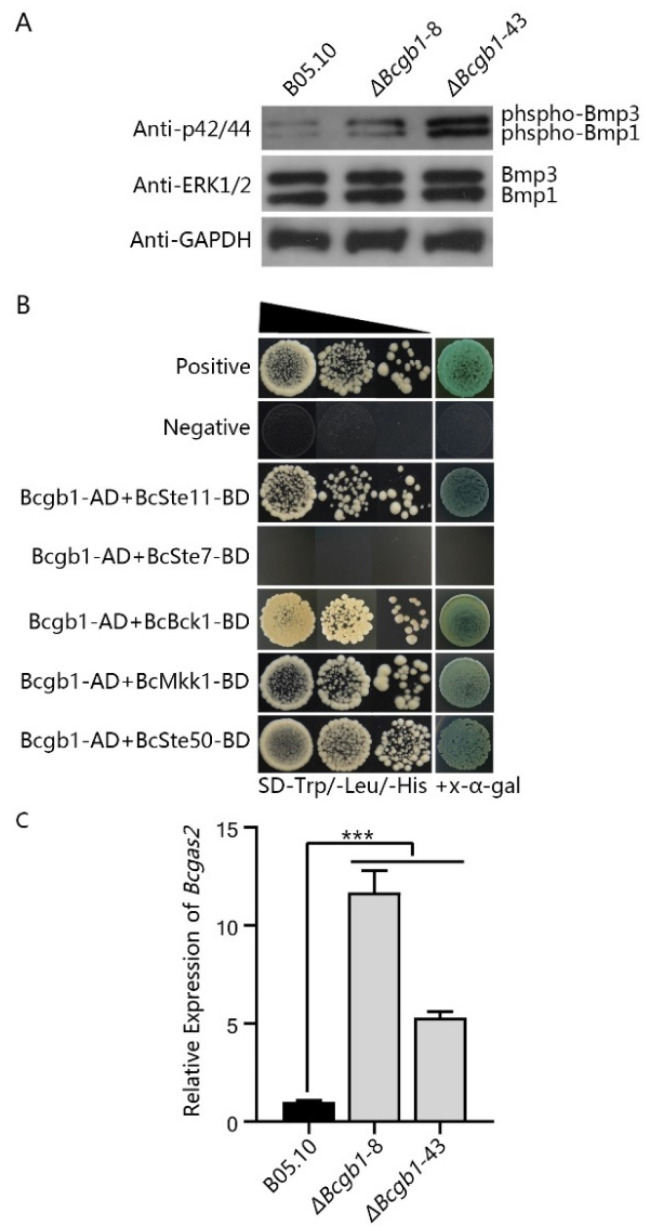
Bcgb1 negatively regulates the Bmp1 and Bmp3 MAPK pathway in *B. cinerea*. (**A**) Phosphorylation level of MAPK (Bmp1 and Bmp3) in the Δ*Bcgb1* mutants. Bmp1 and Bmp3 and their phosphorylated proteins were detected using the ERK1/2 and phospho-p44/42 MAPK antibodies, respectively. The intensity of the phosphorylated Bmp1 and Bmp3 band for each strain is relative to that of the Bmp1 and Bmp3 band, respectively. (**B**) Yeast two-hybrid assay between Bcgb1 and BcSte11/BcSte7/Bmp1 and BcBck1/BcMkk1/Bmp3 cassette. The pGBKT7-53 and pGADT7-T pair of plasmids served as the positive control. The pGBKT7-Lam and pGADT7-T pair of plasmids served as the negative control. Yeast cells were drop-plated on SD-Trp/-Leu/-His with x-α-gal. (**C**) Transcript level of the Bmp1 MAPK-regulated gene *Bcgas2* in the WT and the Δ*Bcgb1* mutants of *B. cinerea*. *** *p* < 0.001.

To investigate whether Bcgb1 plays a role in the MAPK (Bmp1 and Bmp3) signaling pathway, we examined the phosphorylation levels of Bmp1 and Bmp3 in Δ*Bcgb1* mutants with an anti-TpEY antibody. A Western blotting assay showed that Δ*Bcgb1* mutants were increased in Bmp1 and Bmp3 phosphorylation compared with the wild type (Figure 6A). To further explore the role of Bcgb1 in Bmp1 and Bmp3 phosphorylation, we examined the interaction of Bcgb1 with the components of two MAPK signaling cascades (BcSte11/BcSte7/Bmp1, BcBck1/BcMkk1/Bmp3, and the MAPK adapter protein BcSte50). The results of yeast two-hybrid show that Bcgb1 directly interacted with both Bmp1 cascade protein (BcSte11) and Bmp3 cascade proteins (BcBck1 and BcMkk1). Moreover, Bcgb1 directly interacted with the MAPK adapter protein BcSte50 (Figure 6B).

To test whether deletion of Bcgb1 altered expression of the downstream target genes of Bmp1, we measured the transcript level of a target gene, *Bcgas2* [16], in the Δ*Bcgb1* mutants and wild type. The results of the qRT-PCR show that the *Bcgas2* transcript level was significantly increased in the Δ*Bcgb1* mutants (Figure 6C). Our findings suggest that Bcgb1 plays an important role in the MAPK (Bmp1 and Bmp3) signaling pathway.

### 3.7. Deletion of Bcgb1 Affects the Expression of Sclerotia Formation-Related Genes

**Figure 7:**
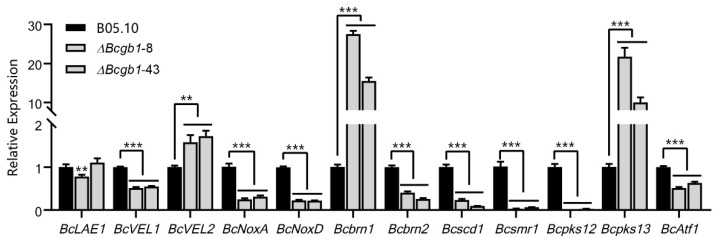
Transcript level of the sclerotia formation-related genes in the WT and the Δ*Bcgb1* mutants of *B. cinerea*. ** *p* < 0.01, *** *p* < 0.001.

Because Δ*Bcgb1* mutants lost the ability to form sclerotia, we examined whether Bcgb1 is involved in controlling the expression of sclerotia formation-related genes in *B. cinerea*. Twelve genes that were confirmed to be related to sclerotia formation were selected to detect the expression in the Δ*Bcgb1* mutants and wild type by qRT-PCR (Figure 7). Three genes encoding the VELVET complex (*BcLaeA1*, *BcVEL1*, and *BcVEL2*) were differentially affected in the Δ*Bcgb1* mutants. The expression of *BcLaeA1* in Δ*Bcgb1* mutants was similar to that in wild type. However, in Δ*Bcgb1* mutants, the transcript level of *BcVEL1* was down-regulated, whereas *BcVEL2* was up-regulated. The expression of two NADPH oxidases genes (*BcNoxA* and *BcNoxD*) was significantly reduced in Δ*Bcgb1* mutants. Among six melanogenic genes, four genes (*Bcbrn2*, *Bcscd1*, *Bcsmr1*, and *Bcpks12*) were repressed in Δ*Bcgb1* mutants. In contrast, other two melanogenic genes (*Bcbrn1* and *Bcpks13*) were overexpressed in Δ*Bcgb1* mutants. Furthermore, expression of the bZIP transcription factor gene *BcAtf1*, which is required for sclerotia formation, was decreased in Δ*Bcgb1* mutants. Taken together, the expression studies suggested that Bcgb1 plays an important role in regulation of sclerotia formation-related gene expression in *B. cinerea*.

## 4. Discussion

In this study, we characterized the function of the Gβ gene *Bcgb1* in *B. cinerea*, which revealed the multifaceted roles of Bcgb1 in development and virulence. To date, the functions of the Gβ gene have already been studied in several plant pathogenic fungi, including *C. parasitica* [24], *M. grisea* [7], *F. oxysporum* [9], *Ustilago maydis* [25], *Cochliobolus heterostrophus* [26], *F. verticillioides* [27], *Gibberella zeae* [28], and *V. dahliae* [12]. Interestingly, the Gβ gene in plant pathogenic fungi played varying roles in development and pathogenicity.

Loss of *Bcgb1* in *B. cinerea* caused mutants with a significant decrease in virulence. This is consistent with the function of the Gβ gene in most plant pathogenic fungi, except that the Gβ gene deletion mutants showed a slightly reduced in virulence in *U. maydis* [25] and *F. verticillioides* [27]. In *B. cinerea*, an infection cushion is a special infection structure that is necessary for successful infection of mycelia. The Δ*Bcgb1* mutant was defective in infection cushion formation, and was responsible for reduced virulence. Similarly, the Gβ gene played a critical role in the infection structure (appressorium) formation and pathogenicity in *M. grisea* [7] and *C. heterostrophus* [26].

Deletion of *Bcgb1* resulted in altered colony morphology and decreased mycelial growth rate, but increased aerial hyphae and mycelia biomass. Alteration of colony morphology was also presented in the Gβ deletion mutant of *F. oxysporum* [9] and *V. dahliae* [12]. In *Aspergillus nidulans*, the Gβ deletion mutant Δ*sfaD* showed a significant reduction in mycelial mass, although the growth rate was similar to wild type [29]. Similar to the Δ*Bcgb1* mutant, more aerial hyphae were also found in the Gβ mutant of *M. grisea* [7]. In contrast, the Gβ gene *cpgb-1* was required for normal aerial hyphae formation in the chestnut blight fungus *C. parasitica* [24]. In *F. verticillioides*, the Gβ gene *gbb1* was dispensable for mycelial growth and mycelial mass but important for mycotoxin fumonisin B_1_ production [27]. These results indicate that Gβ in filamentous fungi plays different roles in mycelial growth.

The Gβ gene is required for sporulation in *B. cinerea*, which was also found in several fungi, such as *C. parasitica* [24], *M. grisea* [7], *F. oxysporum* [9], *C. heterostrophus* [26], and *F. verticillioides* [26]. However, the opposite results, that deletion of Gβ gene caused increased conidiation, were observed in *A. nidulans* [29] and *V. dahliae* [12]. In addition, the Δ*Bcgb1* mutants failed to form sclerotia, but the Gβ mutants of *V. dahliae* enhanced sclerotia formation [12]. It is suggested that the role of Gβ in conidiation and sclerotia formation was opposite in *B. cinerea* and *V. dahliae*. The qRT-PCR results revealed that loss of Gβ affected the expression of sclerotia formation-related genes, indicating that Gβ is an upstream regulatory component of these genes.

In filamentous fungi, G proteins are involved in the regulation of cAMP signaling that controls multiple cellular processes, including growth, development, and virulence [4]. Deletion of the Gβ gene resulted in reduced intracellular cAMP levels in *N. crassa* [30], *M. grisea* [7], and *F. oxysporum* [9]. In addition, loss of Gβ caused a decreased in Gα protein levels in *C. parasitica* [31] and *N. crassa* [30]. Therefore, Gβ should maintain normal levels of Gα protein, which stimulates adenylate cyclase activity to form cAMP [32]. However, the intracellular cAMP levels were drastically increased in Δ*Bcgb1* mutants (Figure 5A), indicating that Gβ serves as an inhibitor to suppress the activity of Gα proteins in *B. cinerea*. The adenylate cyclase (cAMP biosynthesis) and phosphodiesterase (cAMP hydrolysis) are crucial regulators for maintaining the balance of intracellular cAMP levels [33]. In this study, expression of adenylate cyclases gene (*Bac*) and phosphodiesterases genes (*BcPde1* and *BcPde2*) was significantly reduced in the Δ*Bcgb1* mutants (Figure 5B). The possible explanation is that *Bcgb1* deletion inhibits the transcription of *BcPde1* and *BcPde2*, resulting in increased cAMP levels that may feedback suppress the expression of *Bac*. Thus, the activities of adenylate cyclase and phosphodiesterase in Δ*Bcgb1* mutants needs to be further investigated. Another cAMP signaling component is the cAMP-dependent protein kinase (PKA), consisting of two regulatory subunits and two catalytic subunits. In *B. cinerea*, BcPka1 and BcPka2 belong to a catalytic subunit, and BcPkaR is the regulatory subunit [34]. Deletion mutants of PKA (Δ*BcPka1*, Δ*BcPka2*, and Δ*BcPkaR*) all showed significantly increased intracellular cAMP levels in mycelia, suggesting that the PKA (BcPka1, BcPka2, and BcPkaR) negatively regulates the intracellular cAMP levels in *B. cinerea* [34]. Similarly, a significant reduction in expression of three PKA genes (*BcPka1*, *BcPka2*, and *BcPkaR*) and increased intracellular cAMP levels were also observed in Δ*Bcgb1* mutants (Figure 5C).

In *Saccaromyces cerevisiae* yeast, the Gβ protein Ste4p is required to transfer the pheromone signal to activate the MAPK mating pathway [35]. However, deletion of Gβ gene *fgb1* did not affect phosphorylation level of the MAP kinase Fmk1 in *F. oxysporum* [31]. Our results show that phosphorylation levels of MAP kinases (Bmp1 and Bmp3) were increased in Δ*Bcgb1* mutants (Figure 6A), supporting the hypothesis that Gβ regulates the MAPK signaling pathway downstream in *Cryptococcus neoformans* [36] and *M. grisea* [7]. Yeast two-hybrid assays showed that Gβ protein Bcgb1 directly interacted with MAPK cascade proteins (BcSte11, BcBck1, BcMkk1, and BcSte50) (Figure 6B). This provides evidence that Gβ is involved in the MAPK signaling pathway. Additional evidence is that *Bcgas2*, the downstream regulated gene of Bmp1 [16], was up-regulated in the Δ*Bcgb1* mutant. These results suggest that Gβ protein Bcgb1 plays an important role in the regulation of the MAPK signaling pathway in *B. cinerea*.

Previous studies have demonstrated that deletion of the MAP kinase Bmp1 causes defects in conidia germination, reduces mycelial growth, causes a failure to form sclerotia, and induces a loss of pathogenicity in *B. cinerea* [37]. Another MAP kinase, Bmp3, is important for growth, conidiation, sclerotia formation, and virulence [38]. Interestingly, the Δ*Bcgb1* mutants showed similar defective phenotypes, but increased phosphorylation levels of Bmp1 and Bmp3. Maintenance of normal phosphorylation levels of MAPK is critical for the MAPK signaling pathway in eukaryotic cells. Our data indicate that Gβ protein Bcgb1 is required for maintaining normal phosphorylation levels of Bmp1 and Bmp3 in *B. cinerea*.

In conclusion, this study presents evidence that Bcgb1 not only plays an important role in the cAMP signaling pathway, but also regulates the MAPK signaling pathway. Bcgb1 may function in cross-talks between these signaling pathways. This might explain the defects of the Δ*Bcgb1* mutant in mycelial growth, conidiation, sclerotia formation, and virulence. These data provide new insight into the multiple functions of the Gβ protein in filamentous fungi. Further studies are necessary to reveal the molecular mechanism of Gβ in regulating the cAMP signaling pathway and MAPK signaling pathway.

## Data Availability

Not applicable.

## Supplementary Materials

The following are available online at https://www.mdpi.com/article/10.3390/jof7060431/s1, Figure S1: Disruption of *Bcgb1* in *B. cinerea*: (A) Schematic diagram indicating the strategy for disruption of *Bcgb1* by the homologous recombination event in *B. cinerea*. *HPT*, hygromycin phosphate transferase gene, (B) PCR confirmation of disruption of the *Bcgb1* gene in different mutants of *B. cinerea*, and (C) Southern blot confirmation of the disruption of *Bcgb1*; Table S1: Primers used in this study.
