# Supplementary material for: Botrytis cinerea G Protein β Subunit Bcgb1 Controls Growth, Development and Virulence by Regulating cAMP Signaling and MAPK Signaling
Source: J Fungi (Basel). 2021 May 29;7(6):431. doi: 10.3390/jof7060431 (PMC8228952; doi:10.3390/jof7060431)
Supplement: Supplementary file 1 [file jof-07-00431-s001.zip › Table S1.pdf]

Table S1. Primers used in this study

**Primer**	**Sequence(5'-3')**	**Relevant Characteristics**
BcactA	F:TCCAAACCGCCAGTCAATCC<br>R:GATACCACCGCTCTCAAGACC	qRT-PCR detection of the expression of *BcactA* (Liu *et al*., 2011)
BCG1-BD	F:TCAGAGGAGGACCTGCATATGATGGGTTGCGGTA TGAGCACT<br>R:TCGACGGATCCCCGGGAATTCTTAACGCCATGCT GAATTAAG	Amplification of full BCG1 for construction of the vector pGBKT7- BCG1
BCG2-BD	F:TCAGAGGAGGACCTGCATATGATGTGTTTCGGCA GCAGGGAG<br>R:TCGACGGATCCCCGGGAATTCTCAAAGGATCAGC TTTTGAAG	Amplification of full BCG2 for construction of the vector pGBKT7- BCG2
BCG3-BD	F:TCAGAGGAGGACCTGCATATGATGGGTGCTTGCA TGAGCTC<br>R:TCGACGGATCCCCGGGAATTCTTAGAGTATGCCC GAGTCTT	Amplification of full BCG3 for construction of the vector pGBKT7- BCG3
Bcgb1-BD	F:TCAGAGGAGGACCTGCATATGATGTCGTCAAACT CGAACGA<br>R:TCGACGGATCCCCGGGAATTCTTATGTGGACCAG AGTTTGA	Amplification of full bcgb1 for construction of the vector pGBKT7- Bcgb1
Bcgb1-AD	F:CATATGGCCATGGAGGCCAGTATGTCGTCAAACT CGAACGA<br>R:GTATCGATGCCCACCCGGGTGTTATGTGGACCAG AGTTTGA	Amplification of full bcgb1 for construction of the vector pGADT7- Bcgb1
BCGG1-BD	F:TCAGAGGAGGACCTGCATATGATGCCTCAAGGTT ATTCATCT<br>R:TCGACGGATCCCCGGGAATTCTTACATCACCAAA CAGCATCC	Amplification of full BCGG1 for construction of the vector pGBKT7- BCGG1
Bmp1-BD	F:TCAGAGGAGGACCTGCATATGATGACAGCTCGTG CGCCTAAT<br>R:TCGACGGATCCCCGGGAATTCCTATCTCATGATCT CATCATA	Amplification of full Bmp1 for construction of the vector pGBKT7- Bmp1
Bmp3-BD	F:TCAGAGGAGGACCTGCATATGATGGCAGACCTGC AAGGAAG<br>R:TCGACGGATCCCCGGGAATTCCTACGATCGCATA GCATCCA	Amplification of full Bmp3for construction of the vector pGBKT7- Bmp3
Pka1-BD	F:TCAGAGGAGGACCTGCATATGATGCCGACACTCG GTTTTTTG<br>R:TCGACGGATCCCCGGGAATTCTCATCGGGCCCAC CTTGACCA	Amplification of full Pka1 for construction of the vector pGBKT7- Pka1
Pka2-BD	F:TCAGAGGAGGACCTGCATATGATGGCTGAGACCA	Amplification of full Pka2 for

	CGACGGCT<br>R:TCGACGGATCCCCGGGAATTCCTAAAAACCCTTGAAACAATCATCG	construction of the vector pGBKT7- Pka2
Pde1-BD	F:TCAGAGGAGGACCTGCATATGATGGAAGGTAGAGCTCCCGCT<br>R:TCGACGGATCCCCGGGAATTCCTACAAATAAACACTCATCCC	Amplification of full Pde1 for construction of the vector pGBKT7- Pde1
Pde2-BD	F:TCAGAGGAGGACCTGCATATGATGGACTACGCCGCATGCAAC<br>R:TCGACGGATCCCCGGGAATTCTCAACCCGCAGTCCCATCAGA	Amplification of full Pde2 for construction of the vector pGBKT7- Pde2
Bck1-BD	F:TCAGAGGAGGACCTGCATATGATGAGCCAGCGTCCCCAA<br>R:TCGACGGATCCCCGGGAATTCTTAATATGTGCCGCGAATCTTAGC	Amplification of full BcBck1 for construction of the vector pGBKT7- Bck1
BcMkk1-BD	F:TCAGAGGAGGACCTGCATATGATGTCTTCTCCAGCTCCATTA<br>R:TCGACGGATCCCCGGGAATTCTCACCAGCCCCATACGGTAGC	Amplification of full BcMkk1 for construction of the vector pGBKT7- BcMkk1
BcSte50-BD	F:TCAGAGGAGGACCTGCATATGATGGCATTCGAGTCCGGCACA<br>R:TCGACGGATCCCCGGGAATTCTCATATAATGCCGCCAGGAGG	Amplification of full BcSte50 for construction of the vector pGBKT7- BcSte50
BcSte50-AD	F:GTACCAGATTACGCTCATATGATGGCATTCGAGTCCGGCACA<br>R:ATGCCCACCCGGGTGGAATTCTCATATAATGCCGCCAGGAGG	Amplification of full BcSte50 for construction of the vector pGADT7- BcSte50
BcSte11-BD	F:CTGATCTCAGAGGAGGACCTGCATATGATGGCAATGCTAGCTTCAAAG<br>R:TGCAGGTCGACGGATCCCCGGGAATTCTCAAGTGATTGGGTTTAAAAA	Amplification of full BcSte11 for construction of the vector pGBKT7- BcSte11
BcSte7-BD	F:CTGATCTCAGAGGAGGACCTGCATATGATGTCGGACGGTATTGGTTCG<br>R:TGCAGGTCGACGGATCCCCGGGAATTCTTATGAGCCCTGAGCTGCATA	Amplification of full BcSte11 for construction of the vector pGBKT7- BcSte7
BD-jiance	F: ATGGAGGAGCAGAAGCTG<br>R: TGCGGCCGCTGCAGGTCG	PCR detection of recombinant plasmids of pGBKT7
AD-jiance	F: ATGGAGTACCCATACGACGTA<br>R: TCGATGGATCCCGTATCGATG	PCR detection of recombinant plasmids pGA DT7
Bcgas2-RT	F: TCGGTATCGGTGGTCTTTC<br>R: GTACTCAATCGCACGCTTC	qRT-PCR detection of the expression of *Bcgas2*

		(Schamber et al 2010)
BcNoxA-RT	F: ACTGCCCATGCCAAAATTAG<br>R: CCTAAGCAATGACCCCAGAA	qRT-PCR detection of the expression of *BcNoxA* based on *Bcin05g00350*
BcNoxD-RT	F: AAGAATCTCGACGGTGCAGT<br>R: AGCTGTCAAAGGGAGGACAA	qRT-PCR detection of the expression of *BcNoxD* based on *Bcin01g08690*
BcLAE1-RT	F: GATGAGAAAGACCGGATGGA<br>R: TAGCTAGGTCAAGCCCGAGA	qRT-PCR detection of the expression of *BcLAE1* based on *Bcin05g01210*
Bcgb1-L<br><br><br>Bcgb1-R<br><br><br>HY- Bcgb1<br><br><br>YG- Bcgb1	F: TATTCACTGACAATCTCTGGCA<br>R:GCTGCAGGAATTCGATATCAAGTTGCGACCAAATGGATGATAG<br>F:AAAGGAATAGAGTAGATGCCGAATCGCCCTCCCAAATACG<br>R: ATTACAGAGCCTTAGTTATGAGTCC<br>F:CTATCATCCATTTGGTCGCAACTTGATATCGAATTCCTGCAGC<br>R: CTGCTCCATACAAGCCAACC<br>F: TGTCCTGCGGGTAAATAGC<br>R:CGTATTTGGGAGGGCGATTCGGCATCTACTCTATTCCTTT	Gene deletion vector construction of Bcgb1 based on Bcgb1 and KS1004
Bcgb1sy-F	AGAATGACCAAGGCCAAGAGG	PCR detection of *Bcgb1* deletion mutant
HYG	F: GACAGCGTCTCCGACCTGA<br>R: GCTCCATACAAGCCAACCAC	Amplification of part sequence of HPT in the *Bcgb1* deletion mutant
BcVEL1-RT	F: CTTCCTCTTCGCAACTTTGG<br>R: CAACTTGTATTGGCCCTCGT	qRT-PCR detection of the expression of *BcVEL1*
BcVEL2-RT	F: CGAACATGGAATGTTTGTGC<br>R: GTGGCGCACTACCATAGGTT	qRT-PCR detection of the expression of *BcVEL2*
Bcbrn1-RT	F: CCAAGAAGCAATGGGGTAAA<br>R: TCATGATCAAACGACCTCCA	qRT-PCR detection of the expression of *Bcbrn1*
Bcbrn2-RT	F: GACTCTTGAGGGCAAAGTCG<br>R: TGCTACCGAGTTCCTCGATT	qRT-PCR detection of the expression of *Bcbrn2*
Bcscd1-RT	F: TGACAGCTACGATGCAAAGG<br>R: GCCCTCAATTGATGATGTCC	qRT-PCR detection of the expression of *Bcscd1*
Bcsmr1-RT	F: CGTCTCCAGGAAGCTCAATC<br>R: TTTTGTGGCGGAGAGAGAGT	qRT-PCR detection of the expression of *Bcsmr1*
Bcpks12-RT	F: CTGATCTTCGTGGGGTTGTT<br>R: TGCCGCATTGATAGTAGCAG	qRT-PCR detection of the expression of *Bcpks12*
Bcpks13-RT	F: AATGCAGCGTCTGGCTATCT<br>R: CACGCTGTATGATGGACCAC	qRT-PCR detection of the expression of *Bcpks13*

BcAtf1-RT	F: TGTCCCTCCACAAAACAACA<br>R: ACCGGTGAGCATGTTAGGAC	qRT-PCR detection of the expression of *BcAtf1*
Bac-RT	F: CGACTGCGCTTAATACTACCG<br>R: TTATCCTCCCTTCCTACATCTTC	qRT-PCR detection of the expression of *Bac*
BcPde1-RT	F: CTCGCAGACCTCAAACTTCC<br>R: TACTCGCCAATCCAGCAAT	qRT-PCR detection of the expression of *BcPde1*
BcPde2-RT	F: TCACTTCCTCGTCCGCTTAG<br>R: GGGTGACCTACATCGTGGC	qRT-PCR detection of the expression of *BcPde2*
BcPka1-RT	F: TGCACAAGATGAAGCAGGTC<br>R: AGAATTTGGCCACTGGATTG	qRT-PCR detection of the expression of *BcPka1*
BcPka2-RT	F: GCTTGACTACTGCCCTGGAG<br>R: TATGTGTCCCTCAGCATCCA	qRT-PCR detection of the expression of *BcPka2*
BcPkaR-RT	F: CGTCCATGTCAATTCCACTG<br>R: AGGATTCGGCGGAAAGTTAT	qRT-PCR detection of the expression of *BcPkaR*
