# Supplementary figures and images for: Botrytis cinerea G Protein β Subunit Bcgb1 Controls Growth, Development and Virulence by Regulating cAMP Signaling and MAPK Signaling
Source: J Fungi (Basel). 2021 May 29;7(6):431. doi: 10.3390/jof7060431 (PMC8228952; doi:10.3390/jof7060431)

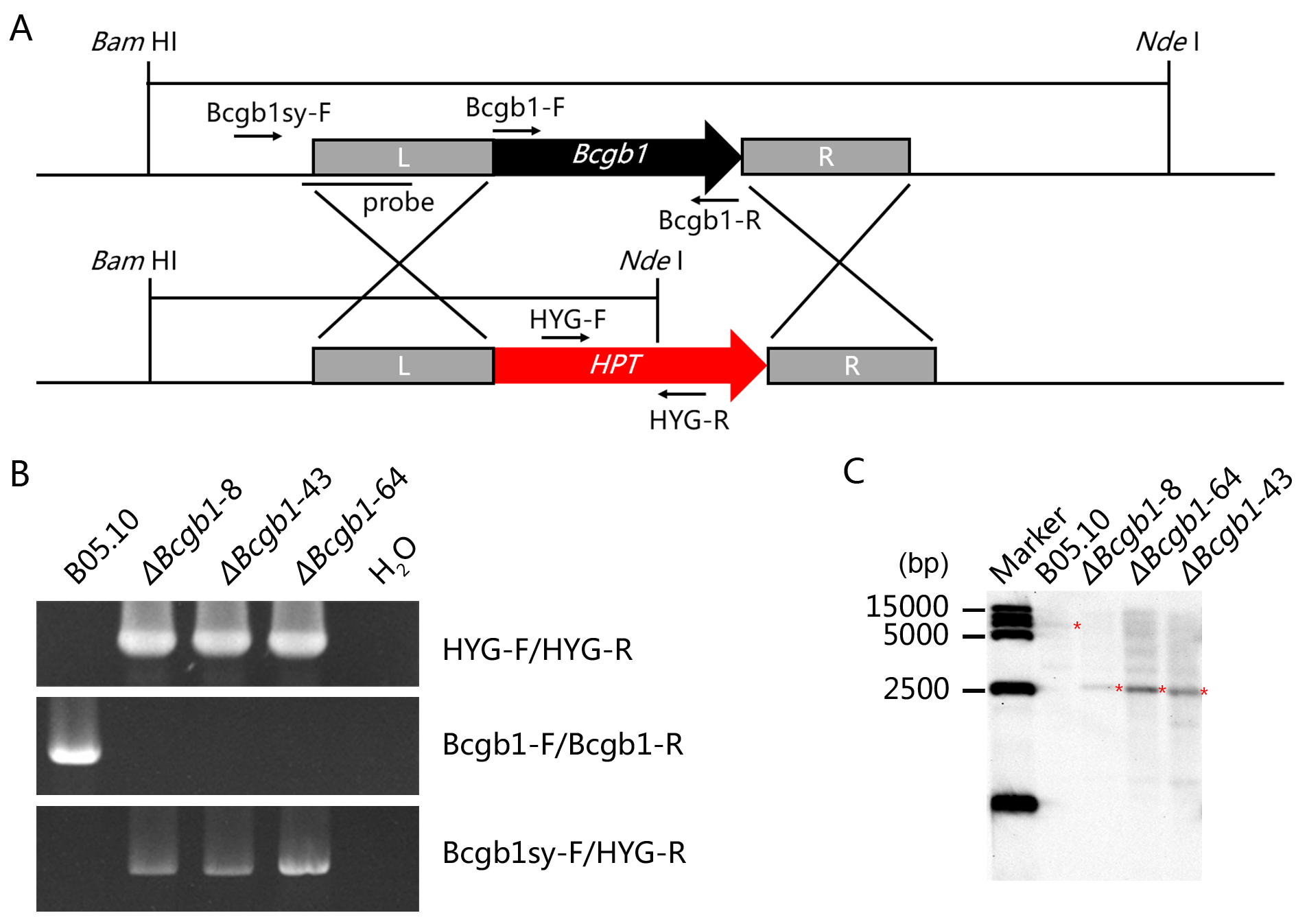

Supplement: Supplementary file 1 [file jof-07-00431-s001.zip › Figure. S1.jpg]
